# Children’s use of psychosocial care in a population-based longitudinal study: less likely for girls, children with a non-Western background and children with a high quality of life

**DOI:** 10.1007/s00787-021-01737-2

**Published:** 2021-03-03

**Authors:** D. G. M. Eijgermans, H. Raat, P. W. Jansen, P. M. van de Looij-Jansen, M. H. J. Hillegers, W. Jansen

**Affiliations:** 1 The Generation R Study Group, Erasmus Medical Center, Rotterdam, the Netherlands; 2 Department of Public Health, Erasmus Medical Center, Rotterdam, the Netherlands; 3 Department of Child and Adolescent Psychiatry/Psychology, Erasmus Medical Center, Rotterdam, the Netherlands; 4 Department of Psychology, Education and Child Studies, Erasmus University Rotterdam, Rotterdam, the Netherlands; 5 Research Department, City of Rotterdam, Rotterdam, the Netherlands; 6 Department of Social Development, City of Rotterdam, PO Box 70032, 3000 LP Rotterdam, the Netherlands

**Keywords:** Mental health, Service utilization, Psychosocial care use, Need for care, School-aged children

## Abstract

Knowledge on determinants of children’s psychosocial care use is important to improve their access to care. This study examined the independent contributions of need and predisposing factors to psychosocial care use in 9-year-old children, guided by the Gateway Provider Model. Data of the Generation R Study, a prospective cohort of children born in Rotterdam, the Netherlands, were analysed using multivariable logistic regression (*n* = 4714). Need (quality of life, presence and type of emotional/behavioural problems) and predisposing factors (sex, ethnic background and maternal educational level) were measured using parent questionnaires at multiple time points between ages 1.5 and 9 years. Psychosocial care use was parent-reported at 9 years old (9.6% among children with Western background, 7.3% among children with non-Western background). Having emotional/behavioural problems at 5 and 9 years old was associated with more care use, while having a higher quality of life, being a girl and having a Moroccan/Turkish or other non-Western background were associated with less care use. Externalising and internalising problems, as well as several types of problems, at 5 and 9 years old were associated with psychosocial care use. Stratified analyses revealed that, in children with non-Western backgrounds, only a poorer psychosocial quality of life was associated with psychosocial care use. To conclude, girls with a Western background and children with a non-Western background were less likely to receive care compared to their peers. Children with parent-reported emotional/behavioural problems at 5 and 9 years old and decreased quality of life at 5 years old were more likely to receive psychosocial care use at 9 years old. Our findings hold relevance for preventive policies.

**Supplementary Information:**

The online version contains supplementary material available at 10.1007/s00787-021-01737-2.

## Background

**Fig. 1:**
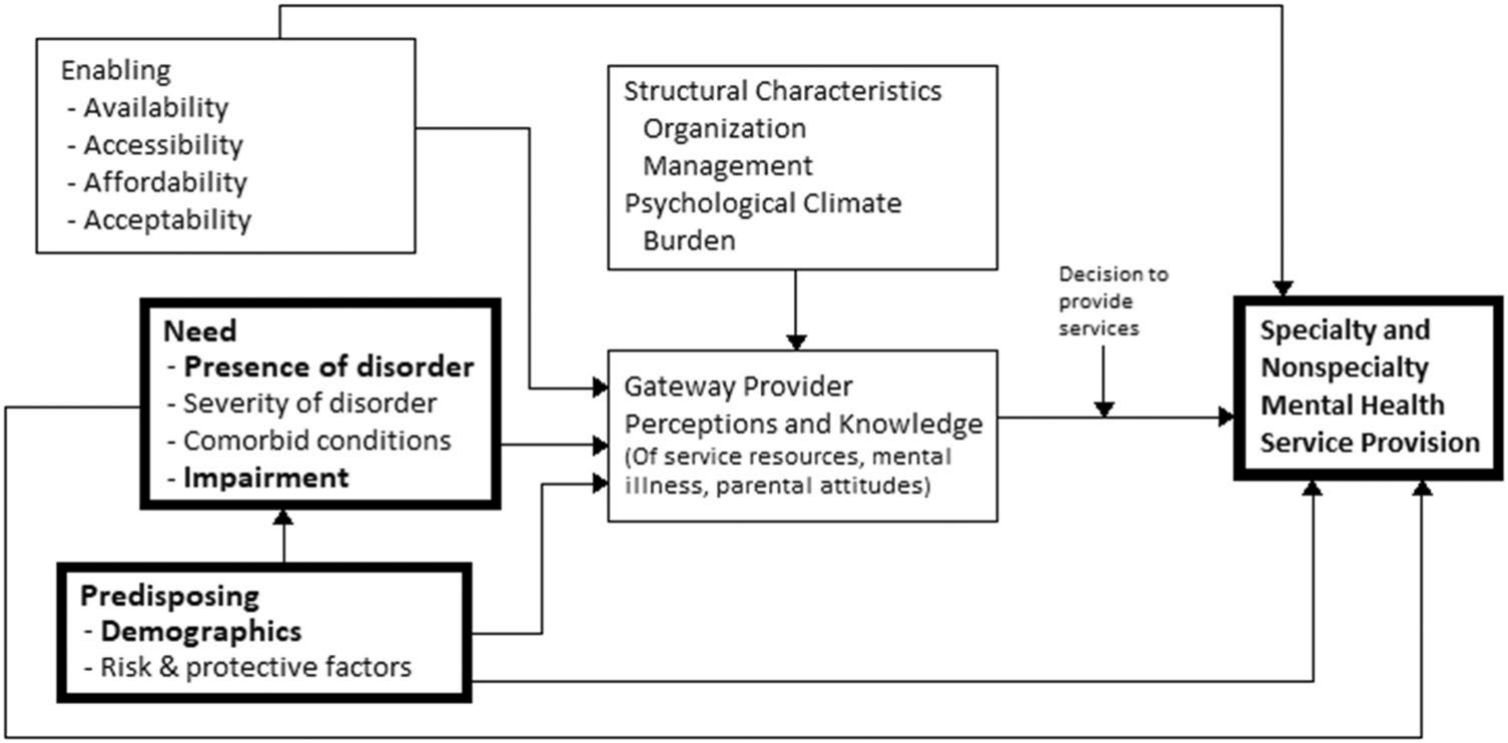
Gateway Provider Model by Stiffman et al. [3]. Reproduced with permission from the principal author Dr. A.R. Stiffman. Determinants addressed in the current paper are printed bold

Psychosocial care use by children has been rising over the past decades in Western countries [1]. Despite this, concerns exist that not all children in need of psychosocial care actually receive this care [1, 2]. To improve access to and delivery of psychosocial care, it is important to improve our understanding of care use determinants. The Gateway Provider Model by Stiffman et al. [3] structures possible determinants of care use. This model is an adjustment of the widely used Behavioural Model of Health Service Use by Andersen et al. [4] and is tailored to the specific situation of children, see Fig. 1. The model suggests that enabling factors (e.g. accessibility, availability), need factors (e.g. presence of mental disorders), and predisposing factors (e.g. demographics) determine children’s psychosocial care use. Furthermore, it suggests that the pathways from these factors to care are influenced by gateway providers (e.g. parents, teachers, general practitioners), and that structural characteristics (e.g. characteristics of the physical environment) influence the provider´s behaviour. In this paper, we focus on predisposing and need factors, some of which are better studied than others.

As expected, multiple studies showed that the presence of emotional/behavioural problems—an indicator of need—is associated with higher psychosocial care use [5, 6]. Less is known about the association with impact of quality of life, although a study by Ford et al. [7] showed an association of poor quality of life with more care use. Findings for types of problems and their association with care use are contradictory on what types of problems are associated with care use. One study found internalising types to be associated with care use [8], another study found externalising types to be associated [5], and again another study found that a mix of internalising and externalising types was associated with care use [9]. Regarding the predisposing factors, it is suggested that children of ethnic minorities are less likely to use psychosocial care compared to children of ethnic majorities, while no differences in the prevalence of problems are detected [6, 10, 11].

Although need and predisposing factors have been studied, the number of longitudinal studies in general populations is low. Assessing determinants early in childhood might be beneficial for improving preventive measures and psychosocial care use by children. Knowing which predisposing and need factors early in life are associated with psychosocial care use later in childhood, can help to target early intervention and prevention strategies towards children and families. This with the ultimate goal to avoid the need for later psychosocial care use. Moreover, little evidence is available on psychosocial care use in children at primary school age and on the independent contribution of the possible determinants on this care use.

This study aims to examine the independent contributions of presence, impact and different types of emotional/behavioural problems as well as sex, maternal ethnic background and maternal educational level on psychosocial care use among 9-year-old children in a population-based cohort. The contribution of need factors (presence of emotional/behavioural problems) is studied at different ages. By doing this, we can identify early prevention and intervention possibilities as well as the main determinants of psychosocial care use.

## Methods

### Study design and population

Generation R Study is a multi-ethnic prospective cohort study of children born between 2002 and 2006 in the city of Rotterdam, the Netherlands, that has been described previously [12]. In this cohort, 9749 children and their parents are followed from fetal life onwards. At the measurement wave at 9 years, 8548 were invited for participation between 2011 and 2014; whereof 5862 visited the research centre. Children were consecutively excluded because of missing data on psychosocial care use at 9 years old (*n* = 358), when not more than one emotional/behavioural problem measure was available (*n* = 733) or when more than 70% was missing on all independent variables (*n* = 57). This resulted in a study sample of 4714 children for the current paper. Children that remained in the study were more likely to be girls, have a Dutch background, and have a mother with a high educational level compared to the original sample. The Medical Ethics Review Board of the Erasmus Medical Center approved the study protocols. All parents provided written informed consent.

### Procedure and measures

Data were retrieved from questionnaires administered to parents when children were 1.5, 3, 5 and 9 years old.

#### Psychosocial care use

Psychosocial care use was measured at 9 years old via the question: ‘Has your child in the past 12 months been examined or treated for any mental health problem by a professional?’ Parents provided answers for eight different psychosocial care providers, namely five specialty mental health care providers, social work, preventive youth health care, and the general practitioner. Also the option ‘other, namely…’ was provided. We recoded preventive youth health care and general practitioners into no psychosocial care use (16% of positive answers) since these organisations mainly examine and refer without treatment, while this study focusses on treatment. All other care providers were combined and categorized as ‘psychosocial care use  in the past year’. A similar question on care use was asked regarding lifetime psychosocial care use.

#### Need factors

Presence, impact and type of emotional/behavioural problems were the need factors studied, in line with the Gateway Provider Model [3]. The Child Behavior Checklist (CBCL) was used to assess presence of overall emotional/behavioural problems and types of emotional/behavioural problems. The CBCL/1.5–5 version was repeatedly assessed up to the age of 5 years old, while the CBCL/6–18 version was used at 9 years old [13]. The CBCL shows good validity and reliability [13]. The CBCL’s were filled out by one of the parents at child ages 1.5 years (81% mother), 3 years (100% mothers), 5 years (92% mother) and 9 years (98% mothers).

A total problem score in the borderline or clinical range was used as an indicator of the *presence* of emotional/behavioural problems. *Type* of problem was assessed as a borderline/clinical score on the specific syndrome scales (seven types for CBCL/1.5–5 and eight for CBCL/6–18 version) and broadband scales for externalising and internalising problems of the CBCL. Dutch norm scores were used for the CBCL/1.5–5 years [14]. International norm scores (based on multiple countries including a Dutch norm sample) were used for the CBCL/6–18 years, which are also used as norm scores for this age group in the Netherlands [15, 16]. The *impact* of emotional/behavioural problems was measured using the Child Health Questionnaire (CHQ) version PF28, a measure of quality of life [17]. The CHQ was completed by one of the parents at the child’s age of 5 years. The CHQ was not available at later ages. The psychosocial and physical summary score of the 28-item CHQ were used and standardized on a 0–100 continuum [17]. A higher score reflects a better quality of life. The CHQ-PH28 shows a high internal consistency, test–retest reliability and good validity [18].

#### Predisposing factors

*Sex* was determined as ‘boy’ or ‘girl’. The mother’s *ethnic background* was used to represent the family’s ethnic background since mothers are generally more involved in raising children than fathers. Moreover, it takes into account the countries of birth of the children’s grandparents too, as opposed to the child’s background. Ethnic background, based on country of birth of both parents of the mother, was obtained via questionnaire and recoded, based on definitions by the Statistics Netherlands [19], into: Dutch, Moroccan/Turkish, Antillean/Surinamese, other Western and other non-Western. These categories were used because they comprise the largest ethnic groups in the Netherlands and typically have similar migration histories. *Educational level* of the mother was obtained via questionnaires when the children were 5 years old. It was recoded in high (higher professional education and university degree), middle (senior general secondary education and secondary vocational education) and low level of education (no education up to pre-vocational secondary education).

### Statistical analyses

Missing data in the independent variables were imputed using multiple imputation methods. Ten imputed datasets were used, equal to the average percentage of missing data over the variables, as based on Van Buuren [20]. Additional variables were included in the imputation process to improve the quality of the imputation, e.g. earlier measures of educational level.

Univariate and multivariable logistic regression analyses were used to determine the associations between need factors, predisposing factors and psychosocial care use. Separate analyses were performed for determinants up to 5 years old and determinants up to 9 years old. In the analyses up to 5 years, the need factors borderline/clinical presence of emotional/behavioural problems and quality of life (impact) were first entered in separate models with the sociodemographic factors and, thereafter, combined into one model. In the analyses up to 9 years, the need factors—borderline/clinical presence of emotional/behavioural problems—were entered together with sociodemographic factors; quality of life was not included. Quality of life was taken into account only in the analyses with need factors up to 5 years old since quality of life was not measured at later ages. In the combined model with analyses up to 5 years, interactions were tested between emotional/behavioural problems (i.e. overall, externalising and internalising problems, and type of problems) and sex, ethnic background, educational level and quality of life. One significant interaction was found at a *p* ≤ 0.01 significance level, namely between presence of problems at 5 years and ethnic background (*p* = 0.007). This suggests a different association between presence of problems and care use for children with a Western and non-Western background. Therefore, stratified analyses were performed for both groups. Ethnic background was dichotomised into Western/non-Western to avoid loss of statistical power due to small groups. To study the associations of psychosocial care use with the syndrome and broadband scales, univariate and multivariable models were used. The multivariable analyses were adjusted for sociodemographic factors. The syndrome/broadband scales were analysed at age 5 and age 9.

As an additional analysis, we studied lifetime (instead of the previous year) psychosocial care use. Furthermore, we performed stratified analyses for gender because of the possible clinical relevance. However, the interaction term of gender with psychosocial problems did not reach significance at *p* ≤ 0.01 level (namely *p* = 0.102).

A significance level of *p* ≤ 0.05 was maintained in all regression analyses, except for the interaction analyses for which the significance level was set at the more conservative level of *p* ≤ 0.01. All multivariable analyses were adjusted for the age of the child at the visit at the research centre. Analyses were performed using IBM SPSS Statistics 24.

## Results

The sample of children consisted of a comparable proportion of boys and girls (49.3% vs 50.7%) with a mean age of 9.8 years (SD: 0.3). Most children had a mother with a Dutch ethnic background (62.6%) and a high educational level (61.3%). The sociodemographic and psychological characteristics of the children are described in Table 1. Psychosocial care was used by 9.0% of the children in the preceding year.

**Table 1:** Characteristics of the total study sample and the subgroups care users and no-care users at 9 years old

Data before imputation	Total study sample (*n* = 4714)		Care users past year (n = 424)		No users past year (*n* = 4290)	
	*n*(%)/Mean(SD)	Miss.%	*n*(%)/Mean(SD)	Miss.%	*n*(%)/Mean(SD)	Miss.%
Psychosocial care use	424 (9.0%)	*0.0*	424 (100%)	*0.0*	0 (0%)	***0.0***
*Predisposing factors*						
Sex						
Boy	2322 (49.3%)	*0.0*	**260 (61.3%)**	*0.0*	**2062 (48.1%)**	*0.0*
Girl	2392 (50.7%)		**164 (38.7%)**		**2228 (51.9%)**	
Age at visit research centre (in years)	9.8 (.3)	*0.0*	9.7 (.3)	*0.0*	9.8 (.3)	*0.0*
Ethnic background mother						
Dutch	2950 (62.6%)	*0.0*	**286 (67.5%)**	*0.0*	**2664 (62.1%)**	*0.0*
Moroccan/Turkish	441 (9.4%)		**28 (6.6%)**		**413 (9.6%)**	
Antillean/Surinamese	368 (7.8%)		36 (8.5%)		332 (7.7%)	
Other Western	595 (12.6%)		53 (12.5%)		542 (12.6%)	
Other non-Western	359 (7.6%)		**21 (5.0%)**		**338 (7.9%)**	
Educational level mother						
High	2747 (61.3%)	*4.9*	242 (59.8%)	*4.5*	2505 (61.5%)	*5.0*
Middle	1308 (29.2%)		130 (32.1%)		1178 (28.9%)	
Low	426 (9.5%)		33 (8.1%)		393 (9.6%)	
*Need factors*						
Presence of overall emotional/behavioural problems						
Present at 1.5 years	304 (8.4%)	*23.2*	**40 (12.3%)**	*23.6*	**264 (8.0%)**	*23.2*
Present at 3 years	224 (6.2%)	*23.4*	**34 (10.5%)**	*23.3*	**190 (5.8%)**	*23.4*
Present at 5 years	295 (6.3%)	*5.0*	**70 (17.2%)**	*4.2*	**225 (5.2%)**	*5.1*
Present at 9 years	400 (9.3%)	*8.7*	**124 (29.2%)**	*9.7*	**276 (7.0%)**	*8.6*
Quality of life at age 5 years (impact score)						
Psychosocial score	53.0 (6.7)	*18.0*	**48.9 (7.7)**	*22.2*	**53.4 (6.5)**	*17.6*
Physical score	57.3 (6.2)	*18.0*	**56.6 (7.7)**	*22.2*	**57.4 (6.1)**	*17.6*

Percentages are the valid percentages and, therefore, add up to 100% without the missing values. Miss. = missingness within the variable. Bold: represents a significant difference between care users in the past year and no-care users in the past year (*p* ≤ .05)

### Need factors

The need factors presence of emotional/behavioural problems at all ages and a poor quality of life were significantly associated with more psychosocial care use at 9 years old in the univariate analyses (*p* ≤ 0.05, see Table 2). In the multivariable analyses with determinants up to 5 years old, children with problems at 5 years old had significantly higher odds (odds ratio (OR): 3.41, 95% confidence interval (95% CI):2.48–4.69) of psychosocial care use compared to children without problems when adjusted for sociodemographic factors. One point (on a 0–100 scale) increase in psychosocial quality of life lowered the odds for care use (OR 0.96, 95% CI 0.95–0.97), while physical quality of life showed the reverse association (OR 1.02, 95% CI 1.00–1.03). In the combined model with both presence of emotional/behavioural problems and quality of life, presence and psychosocial quality of life showed a significant association. The odds ratios of quality of life remained stable, while the odds ratios of the presence of emotional/behavioural problems at 5 years old decreased (Table 2).

**Table 2:** Associations between need factors up to 5 years old, predisposing factors and psychosocial care use at 9 years old (*n* = 4714)

Independent variables	Univariate	Multivariable analysis: presence of problems	Multivariable analysis: quality of life	Multivariable analysis: presence of problems and quality of life
	OR (95% CI)	OR (95% CI)	OR (95% CI)	OR (95% CI)
*Need factors*				
Presence of overall emotional/behavioural problems^a^ (yes)				
1.5 years	**1.60 (1.13–2.27)**	1.23 (0.83–1.82)	*n.a*	1.22 (0.82–1.82)
3 years	**1.88 (1.29–2.75)**	1.25 (0.81–1.94)		1.22 (0.78–1.89)
5 years	**3.57 (2.68–4.77)**	**3.41 (2.48–4.69)**		**2.71 (1.94–3.78)**
Quality of life at 5 years^b^ (impact score)				
Psychosocial quality of life	**0.97 (0.97–0.98)**	n.a	**0.96 (0.95–0.97)**	**0.97 (0.95–0.98)**
Physical quality of life	**0.99 (0.98–1.00)**		**1.02 (1.00–1.03)**	1.01 (1.00–1.02)
*Predisposing factors*				
Sex				
Boy (ref.)	1.00	1.00	1.00	1.00
Girl	**0.58 (0.48–0.72)**	**0.61 (0.50–0.74)**	**0.60 (0.48–0.73)**	**0.62 (0.50–0.76)**
Ethnic background mother				
Dutch (ref.)	1.00	1.00	1.00	1.00
Moroccan/Turkish	**0.63 (0.43–0.93)**	**0.50 (0.32–0.77)**	**0.55 (0.36–0.85)**	**0.47 (0.30–0.73)**
Antillean/Surinamese	1.01 (0.94–1.09)	0.82 (0.56–1.21)	0.83 (0.57–1.22)	0.74 (0.50–1.09)
Other Western	0.91 (0.68–1.21)	0.88 (0.64–1.20)	0.88 (0.64–1.20)	0.84 (0.61–1.15)
Other non-Western	**0.58 (0.46–0.73)**	**0.49 (0.30–0.78)**	**0.50 (0.31–0.80)**	**0.45 (0.28–0.72)**
Educational level mother				
High (ref.)	1.00	1.00	1.00	1.00
Middle	1.14 (0.91–1.42)	1.17 (0.93–1.48)	1.17 (0.92–1.48)	1.13 (0.89–1.43)
Low	0.88 (0.60–1.29)	0.97 (0.64–1.48)	0.93 (0.62–1.41)	0.89 (0.58–1.34)

Bold: represents *p* ≤ 0.05. *OR*  Odds ratio for psychosocial care use at age 9 years, *95% CI*  95% confidence interval. ^a^Total problem score on Child Behavior Checklist in the borderline or clinical range. ^b^Score on Child Health Questionnaire (ranging from 0–100). Adjusted for ‘Age at visit research centre’

In the multivariable model with determinants up to 9 years old (see Table 3), emotional/behavioural problems at 9 years showed the highest OR of the need factors in the multivariable analyses (OR 4.86, 95% CI 3.72–6.35). Problems at 5 years old remained significant when adjusted for problems at 9 years (OR 1.91, 95% CI 1.35–2.70).

**Table 3:** Associations between need factors up to 9 years old and psychosocial care use at 9 years old, adjusted for predisposing factors (*n* = 4714)

Independent variables	Univariate analysis	Multivariable analysis^a^
	OR (95% CI)	OR (95% CI)
Presence of overall emotional/behavioural problems (yes)^b^		
1.5 years	**1.60 (1.13–2.27)**	1.09 (0.73–1.64)
3 years	**1.88 (1.29–2.75)**	1.10 (0.70–1.72)
5 years	**3.57 (2.68–4.77)**	**1.91 (1.35–2.70)**
9 years	**5.59 (4.68–7.61)**	**4.86 (3.72–6.35)**

Bold: represents *p* ≤ 0.05. *OR* Odds ratio for psychosocial care use at age 9 years, *95% CI*   95% confidence interval. ^a^Adjusted for sex, ethnic background mother, educational level mother and age at visit research centre. ^b^Total problem score on Child Behavior Checklist in the borderline or clinical range

### Predisposing factors

In all analyses, sex and ethnic background were associated with psychosocial care use, but maternal educational level was not (Table 2). The results indicated that girls were less likely to receive care than boys (OR 0.62, 95% CI 0.50–0.76). Moroccan/Turkish (OR 0.47, 95% CI 0.30–0.73) and other non-Western children (OR 0.45, 95% CI 0.28–0.72) were less likely to receive care than Dutch children.

### Analyses stratified in subgroups: Western and non-Western children

As a significant interaction was found between ethnic background and the borderline/clinical presence of emotional/behavioural problems at 5 years old (*p* = 0.007), results were stratified for Western and non-Western children. Table 4 shows that in children with a Western background (9.6% psychosocial care use), presence of problems at 5 years old (OR 3.55, 95% CI 2.40–5.24) was associated with psychosocial care use. Furthermore, a better psychosocial (OR 0.97, 95% CI 0.95–0.98) and being a girl (OR 0.56, 95% CI 0.44–0.70) were associated with using less psychosocial care in the Western stratum. In children with a non-Western background (7.3% psychosocial care use), only psychosocial quality of life (OR 0.97, 95% CI 0.95–1.00) was associated with psychosocial care use. Presence of emotional/behavioural problems (at 5 years (OR 1.41, 95% CI 0.73–2.70)) and sex (OR 0.91, 95% CI 0.58–1.43) were not associated.

**Table 4:** Associations between need factors, predisposing factors and psychosocial care use at 9 years old, stratified by ethnic background

Independent variables	Multivariable analysis, stratum: Western (*n* = 3545, care use * n* = 339 (9.6%))	Multivariable analysis, stratum: non-Western (*n* = 1169, care use * n* = 85 (7.3%))
	OR (95% CI)	OR (95% CI)
*Need factors*		
Presence of overall emotional/behavioural problems^a^ (yes)		
1.5 years	1.40 (0.88–2.25)	0.90 (0.44–1.87)
3 years	1.18 (0.67–2.06)	1.66 (0.81–3.40)
5 years	**3.55 (2.40–5.24)**	1.41 (0.73–2.70)
Quality of life at 5 years^b^ (impact score)		
Psychosocial	**0.97 (0.95–0.98)**	**0.97 (0.95–1.00)**
Physical	1.01 (1.00–1.03)	1.01 (0.98–1.00)
*Predisposing factors*		
Sex		
Boy (ref.)	1.00	1.00
Girl	**0.56 (0.44–0.70)**	0.91 (0.58–1.43)
Educational level mother		
High (ref.)	1.00	1.00
Middle	1.08 (0.82–1.42)	1.39 (0.81–2.39)
Low	0.82 (0.48–1.39)	1.03 (0.53–2.01)

Bold: represents *p* ≤ 0.05. *OR*  Odds ratio for psychosocial care use at age 9 years, *95% CI *  95% confidence interval. ^a^Total problem score on Child Behavior Checklist in the borderline or clinical range. ^b^Score on Child Health Questionnaire (ranging from 0 to 100). Adjusted for age at visit research centre. Interaction term ‘presence of emotional/behavioural problems at 5 years*dichotomised ethnic background’, added to the multivariable analysis of Table 2, had a *p* value of 0.007

In Western children, four types of problems at 5 years old were associated with psychosocial care use at 9 years old, i.e. emotionally reactive, withdrawn, attention and aggressive problems (Table 5). Three types of problems at 9 years old were associated with care use, i.e. anxious/depressed, thought problems and attention problems. In both analyses, we adjusted for the other types of problems and sociodemographic factors. Externalising and internalising problems were both at 5 and 9 years old independently associated with care use. In non-Western children, none of the types at 5 years old was associated, and only thought problems (OR 2.34, 95% CI 1.01–5.46) at 9 years old was associated with care use. Internalising problems at 5 years old (OR 1.84, 95% CI 1.03–3.30) and externalising problems at 9 years old (OR 3.22, 95% CI 1.64–6.33) were associated in non-Western children (Table 5). The non-stratified analyses can be found in Appendix (Table SI).

**Table 5:** Associations between types of emotional/behavioural problems or externalising and internalising problems at 5 and 9 years old, and psychosocial care use at 9 years old, stratified by ethnic background (*n* = 4714)

	Multivariable analysis^a^, stratum: Western (*n* = 3545, care use *n* = 339 (9.6%))	Multivariable analysis^a^, stratum: non-Western (*n* = 1169, care use *n* = 85 (7.3%))
	OR (95% CI)	OR (95% CI)
*Type of problem*^*b*^* at age 5 years old*		
Emotionally reactive	**1.75 (1.08–2.85)**	0.72 (0.28–1.85)
Anxious depressed	1.27 (0.85–1.92)	1.67 (0.87–3.19)
Somatic complaints	1.41 (0.98–2.02)	1.06 (0.60–1.90)
Withdrawn	**1.33 (1.02–1.73)**	1.17 (0.69–2.00)
Sleep problems	1.14 (0.80–1.62)	0.92 (0.51–1.67)
Attention problems	**1.80 (1.31–2.49)**	0.86 (0.44–1.68)
Aggressive problems	**1.82 (1.02–3.25)**	2.80 (0.95–8.26)
Externalising scale	**2.19 (1.50–3.21)**	1.03 (0.48–2.25)
Internalising scale	**2.12 (1.52–2.95)**	**1.84 (1.03–3.30)**
*Type of problem*^*b*^* at age 9 years old*		
Anxious/depressed	**2.88 (1.83–4.54)**	1.34 (0.50–3.58)
Withdrawn/depressed	1.45 (0.97–2.17)	1.97 (0.86–4.50)
Somatic complaints	1.28 (0.82–2.00)	0.49 (0.20–1.16)
Social problems	1.45 (0.86–2.46)	0.92 (0.36–2.35)
Thought problems	**1.75 (1.14–2.70)**	**2.34 (1.01–5.46)**
Attention problems	**3.91 (2.67–5.53)**	2.20 (0.86–5.63)
Rule breaking	1.06 (0.49–2.29)	1.15 (0.33–3.96)
Aggressive behaviour	1.57 (0.93–2.65)	1.11 (0.33–3.75)
Externalising scale	**3.07 (2.21–4.26)**	**3.22 (1.64–6.33)**
Internalising scale	**3.05 (2.30–4.04)**	1.69 (0.94–3.05)

Bold:represents *p* ≤ 0.05. *OR* Odds ratio for psychosocial care use at age 9 years, *95% CI* 95% confidence interval. ^a^Adjusted for the other syndrome/broadband scales, sex, ethnic background mother, educational level mother, age at visit research centre. ^b^The problems represent the presence of borderline/clinical problems on the syndrome/broadband scales of the Child Behavior Checklist. The syndrome scales and broadband scales are analysed in separate models. Interaction term ‘presence of emotional/behavioural problems at 5 years *dichotomised ethnic background’, added to the multivariable analysis of Table 2, had a *p* value of 0.007

### Additional analyses

The analysis with lifetime psychosocial care use showed comparable results with the analysis of care use in the past year at age 9 (Appendix, Table SII). The only difference in the multivariable analysis is that all children with a non-Dutch ethnic background showed a significantly decreased odds on psychosocial care use (as opposed to no association for children with an Antillean/Surinamese and other Western background in the analysis with care use in the past year).

In the exploratory analyses, results were stratified based on gender. Among boys, results were comparable to the results of the total sample, except that having a mother with middle educational level showed a positive association and having an Antillean/Surinamese background showed a negative association with psychosocial care use. Among girls, only psychosocial quality of life at 5 years was associated with care use; presence of overall problems and ethnic background were not associated. For types of problems, only internalising problems at 5 years old and anxious/depressed, attention, externalising and internalising problems at 9 years old were associated with care use in girls (See supplementary tables SIII and SIV).

## Discussion

In line with the Gateway Provider Model, we found associations of different need and predisposing factors with psychosocial care use at 9 years old. In the stratified analyses, important differences were identified between children with a Western and a non-Western background. In children with a Western background, having emotional/behavioural problems (parent-reported), a poor quality of life and being a boy were all independently associated with psychosocial care use. However, in children with a non-Western background, only a poor psychosocial quality of life was associated with later psychosocial care use. In the non-stratified analyses, having emotional/behavioural problems and a poor quality of life (impact) at 5 years old were independently associated with psychosocial care use. Having emotional/behavioural problems, i.e. overall problems, and externalising and internalising problems, at 5 and 9 years was associated with psychosocial care use. Also, having a poorer psychosocial quality of life at 5 years old was associated with care use. Moreover, the likelihood of receiving psychosocial care was lower for girls and children with specific non-Western backgrounds. Educational level of the mother was not associated with care use. Although the interaction term regarding gender was not significant, the stratified analyses hinted at possible differences between boys and girls. Findings for boys were similar to the non-stratified analyses, whereas findings for girls showed that less determinants for psychosocial care use were identified in comparison with boys.

According to the parent reports, 9.0% of all children in this sample received psychosocial care in the previous year, as measured at 9 years old (measuring period 2011–2014). Despite the relatively healthy sample we studied, this percentage is comparable to registered care use of 2015 in Rotterdam (10.3%) and in the Netherlands (10.0%) [21].

Previous studies showed that having a minority background is a predisposing factor for less use of psychosocial care [6, 10, 22]. In agreement with Bevaart et al. [6] and De Haan et al. [22], our results also point at lower care use by Moroccan/Turkish and other non-Western children compared to Dutch children. A possible explanation for this might be that non-Western parents tend to experience more barriers towards psychosocial care, e.g. greater stigma [23]. Furthermore, non-Western parents may tend to perceive emotional/behavioural problems as less severe as compared to Western parents [24, 25]. This is in line with our findings on the broadband scales; in Western children, both externalising and internalising problems were associated with care use and, in non-Western children, internalising problems were associated at 5 years old and externalising problems associated at 9 years old.

Regarding the predisposing factor sex, our study’s finding that girls are less likely to receive psychosocial care than boys—unrelated to their level of emotional/behavioural problems—is in line with several other North-Western European studies in comparable age categories [6, 9, 26]. Interestingly, Laitinen-Krispijn et al. [9] and Raven et al. [26] showed that this association reverses as children become older, as during adolescence, girls are more likely to receive care than boys. This might be explained by the different ages of onset of internalising and externalising problems and the unequal distribution of these problems between boys and girls [26]. As we controlled for emotional/behavioural problems, this explanation seems to only hold partly within our study population and calls for further study to explain this sex difference. Our exploratory analyses seem to indicate that in boys more types of problems are associated with care use than in girls. The predisposing factor maternal educational level was not associated with care use, similar to other studies [5, 6]. An explanation might be that maternal educational level and income are closely related and that psychosocial care is free for children in the studied countries.

Overall, externalising, internalising and several specific problem types at 9 years old were associated with psychosocial care use at 9 years old. Since this part of our research is cross-sectional, no conclusions can be drawn on causality. Moreover, caution in the interpretation of these results is needed as psychosocial care use at 9 years old was reported over the preceding year, while parent-reported problems at 9 years old were reported over the preceding 6 months. Based on our data, we cannot determine if and how this influenced our findings, e.g. reverse causality. Moreover, children who already show problems and a decreased quality of life at 5 years old are more likely to receive care at the 9 years old. These findings are of interest for early intervention purposes [27]. Furthermore, both externalising and internalising problems at age 5 and 9 years showed significant associations with psychosocial care use.

Regarding types of problems, we found that withdrawn behaviour, attention problems and aggressive problems at 5 years old were associated with increased care use. At 9 years old, anxious/depressed, withdrawn/depressed, thought and attention problems were associated with care use. Stratified analyses on gender and ethnic background show potentially specific determinants for these subgroups. Other studies—investigating types of emotional/behavioural problems based on the CBCL syndrome scales in association with psychosocial care use—found different types of problems to be associated. Laitinen-Krispijn et al. [9] reported associations with withdrawn and delinquent behaviour, Pihlakoski et al. [5] with destructive behaviour, and Zwaanswijk et al. [8] with anxious/depressed behaviour and thought problems. Our results confirm the findings of most of these studies. We only found no evidence for delinquent and destructive behavior (CBCL scale rule-breaking behavior), which might be explained by the young age of our study population, with 9-years old children typically not engaging in stealing and vandalism yet. More research is warranted into the associations between type of problems and psychosocial care use and the role of age, gender and ethnic background herein.

We studied quality of life as an indicator of impact, to our knowledge an understudied topic [7, 28]. According to the model by Wilson and Cleary [29], quality of life and impact are closely related. In our stratified analyses, a poor psychosocial quality of life (high impact) was a factor that was associated with care use in all children (Western and non-Western). Previous studies demonstrate that if mental problems have an impact on an individual’s or his/her family’s life, the likelihood of receiving psychosocial care increases [7, 30]. Quality of life might, therefore, also be a mediator in the association between emotional/behavioural problems and psychosocial care use. Our results in Table 2 indicate that psychosocial quality of life and emotional/behavioural at 5 years old are independently associated with psychosocial care use. In these analyses, psychosocial quality of life attenuates the effect of emotional/behavioural problems. An additional analysis showed an association between emotional/behavioural problems and psychosocial quality of life (β  − 11.30, 95% CI  − 11.08 to   − 7.79), with which the criteria of Baron, Kenny [31] for partial mediation would be met. The same criteria are met for the types of problems at 5 years old that were significantly associated with care use (data not shown). However, for non-Western children our findings do not support this possible mechanism.

### Strengths and limitations

This study has several strengths. First, we used a large general population sample, where other studies focused on children with clinical problems only [10]. Studying the general population is important because also children without emotional/behavioural problems or with subclinical problems use psychosocial care [2]. A second strength is the longitudinal design. We performed separate analyses, where all independent variables were measured before the assessment of psychosocial care use at age 9 years, which decreased the likelihood that psychosocial care influenced the perceived need. A third strength is that we focused on children instead of adolescents; determinants of mental health care use by adolescents have been studied more frequently as can be seen in the systematic review by Zwaanswijk et al. [28].

This study also has some limitations. First, the drop-out within the Generation R Study might decrease the representativeness of the sample [12]. Therefore, the results of this study should be generalised with caution. We have no reason, however, to believe that this drop-out has influenced our study findings as the percentage of care users was comparable to registry data and sociodemographic factors were accounted for. A second limitation is that caution is needed in the interpretation of the stratified analyses because of potential power issues, especially in the non-Western stratum. Third, we used an observational design, and, therefore, conclusions about causal relationships are limited. Lastly, the variables were measured via parent-reported questionnaires, so all used data is proxy-reported. Furthermore, using questionnaires might have introduced recall bias and, consequently, an underestimation of the outcome seems most likely [32]. People are probably more likely to forget care was obtained than to over-report care use.

### Future research and implications

Additional research is needed for a better understanding of sex and ethnic differences—regardless of emotional/behavioural problems—in psychosocial care use to improve access to and delivery of care. Also, more research is needed to unravel the association between types of problems and care use since the current literature is contradictory. We recommend studying these topics in various, large, longitudinal datasets. Moreover, the additional analyses showed that specific subgroups may receive less care and may have different determinants of psychosocial care use. More research is needed to unravel causal pathways behind these differences. Understanding these pathways contributes to our understanding of lower psychosocial care use among girls and children with a non-Western background.

Our findings suggest that gateway providers of children, e.g. (child) health professionals, teachers and general practitioners, should be aware of the lower use of psychosocial care in Western girls and children with non-Western backgrounds compared to boys and children with a Western background, irrespective of problem levels. Furthermore, psychosocial quality of life at 5 years old—indicating the impact of problems—was a stable factor associated with care use. Therefore, it is important to focus on impact of problems on children’s psychosocial quality of life for early identification and for preventive policies.

## Conclusion

Our findings indicate that generally, the presence of parent-reported emotional/behavioural problems, i.e. overall problems, externalising and internalising problems, and several types of problems, as well as a poor psychosocial quality of life at 5 and 9 are associated with psychosocial care use. Yet, children with specific non-Western backgrounds and—within the group of children with a Western background—girls are less likely to receive psychosocial care at 9 years old, irrespective of the level of emotional/behavioural problems. In particular, we observed that parent-reported emotional/behavioural problems at 5 and 9 years old are associated with psychosocial care use in Western children, but not in non-Western children. Poor psychosocial quality of life (high impact) at 5 years is associated with care use in all children. These findings indicate room to improve access to and delivery of care to reduce disparities in psychosocial care use, especially for girls and non-Western children. Further research is warranted to improve our understanding of determinants of psychosocial care use in children.

## Supplementary Information

Below is the link to the electronic supplementary material.

Supplementary file1 (DOCX 20 KB)

## Data Availability

See the Generation R design paper [12]. Data are available when collaboration is established.
