# Supplementary material for: Children’s use of psychosocial care in a population-based longitudinal study: less likely for girls, children with a non-Western background and children with a high quality of life
Source: Eur Child Adolesc Psychiatry. 2021 Mar 3;31(7):1–11. doi: 10.1007/s00787-021-01737-2 (PMC9343259; doi:10.1007/s00787-021-01737-2)
Supplement: Supplementary file 1 — Supplementary file1 (DOCX 20 KB) [file 787_2021_1737_MOESM1_ESM.docx]

# Appendix

Appendix Table S I, Associations between types of emotional/behavioural problems or externalising and internalising problems at 5 and 9 years old, and psychosocial care use at 9 years old (N=4,714)

|  | Multivariable analysis^a^ |
| --- | --- |
| *Type of problem at age 5 years old* | *OR (95% CI)* |
| Emotionally reactive  Anxious depressed  Somatic complaints  Withdrawn  Sleep problems  Attention problems  Aggressive problems | 1.38 ( .91-2.11)  1.36 ( .96-1.92)  1.28 ( .95-1.74)  **1.32 (1.04-1.66)**  1.05 ( .77-1.44)  **1.51 (1.13-2.02)**  **2.04 (1.24-3.37)** |
| Externalising scale  Internalising scale | **1.83 (1.30-2.58)**  **2.03 (1.52-2.71)** |
| *Type of problem at age 9 years old* | *OR (95% CI)* |
| Anxious/depressed  Withdrawn/depressed  Somatic complaints  Social problems  Thought problems  Attention problems  Rule breaking  Aggressive behaviour | **2.56 (1.72-3.84)**  **1.53 (1.07-2.19)**  .93 ( .63-1.37)  1.21 ( .77-1.89)  **1.82 (1.24-2.66)**  **3.55 (2.50-5.06)**  1.04 ( .55-1.99)  1.49 ( .93-2.40) |
| Externalising scale  Internalising scale | **3.07 (2.29-4.12)**  **2.69 (2.09-3.46)** |

Bold: represents p≤0.05. OR=Odds Ratio for psychosocial care use at age 9 years. 95% CI=95% confidence interval. ^a^Adjusted for the other syndrome/broadband scales, sex, ethnic background mother, educational level mother, age at visit research centre. The problems represent the presence of borderline/clinical problems on the syndrome/broadband scales of the Child Behavior Checklist. The syndrome scales and broadband scales are analysed in separate models.

Appendix Table S II, Associations between need factors, predisposing factors and lifetime psychosocial care use, non-stratified (N=4,714)

|  | Univariate analysis | Multivariable analysis as in Table 2 |
| --- | --- | --- |
|  | *OR (95% CI)* |  |
| Presence of overall emotional/ behavioural problems^a^ (yes) | | |
| 1.5 years  3 years  5 years | **1.59 (1.18-2.15)**  **2.05 (1.49-2.82)**  **3.77 (2.92-4.88)** | 1.20 ( .84-1.70)  1.35 ( .92-1.98)  **2.78 (2.06-3.74)** |
| Quality of life at 5 years^b^ (impact score)  Psychosocial | **.97 ( .96- .98)** | **.96 ( .95- .97)** |
| Physical | **.99 ( .99-1.00)** | **1.02 (1.01-1.03)** |
| Sex  Boy (ref.)  Girl | 1.00  **.59 ( .50- .70)** | 1.00  **.63 ( .53- .75)** |
| Ethnic background mother  Dutch (ref.)  Moroccan/Turkish  Antillean/Surinamese  Other Western  Other non-Western | 1.00  **.63 ( .45- .87)**  .89 ( .66-1.20)  **.75 ( .57- .98)**  **.63 ( .52- .75)** | 1.00  **.50 ( .34- .73)**  **.67 ( .48- .95)**  **.68 ( .51- .90)**  **.50 ( .34- .74)** |
| Educational level mother  High (ref.)  Middle  Low | 1.00  1.04 ( .86-1.25)  .73 ( .51-1.06) | 1.00  1.02 ( .83-1.25)  .71 ( .48-1.05) |

Bold: represents p≤0.05. OR=Odds Ratio for psychosocial care use at age 9 years. 95% CI=95% confidence interval. ^a^Total problem score on Child Behavior Checklist in the borderline or clinical range. ^b^Score on Child Health Questionnaire (ranging from 0-100). The number of lifetime care use is 629 (13,3%) children. Adjusted for Age at visit research centre.

Appendix Table S III, Associations between need factors, predisposing factors and psychosocial care use at 9 years old, stratified by gender

|  | Multivariable analysis, stratum: Boys  (N=2,322, care use N=260 (11.1%)) | Multivariable analysis, stratum: Girls  (N=2,392, care use N=164 (6.9%)) |
| --- | --- | --- |
| *Independent variables* | *OR (95% CI)* | *OR (95% CI)* |
| *Need factors* |  |  |
| Presence of overall emotional/behavioural problems^a^ (yes) | | |
| 1.5 years | .95 ( .55-1.63) | 1.80 ( .99-3.29) |
| 3 years | 1.35 ( .79-2.32) | .91 ( .41-2.00) |
| 5 years | **3.38 (2.24-5.10)** | 1.73 ( .95-3.17) |
| Quality of life at 5 years^b^ (impact score) | |  |
| Psychosocial | **.97 ( .95- .98)** | **.97 ( .95- .99)** |
| Physical | 1.01 (1.00-1.03) | 1.01 ( .99-1.03) |
| *Predisposing factors*  Ethnic background mother  Dutch (ref.)  Moroccan/Turkish  Antillean/Surinamese  Other Western  Other non-Western | 1.00  **.32 ( .17- .58)**  **.56 ( .33- .96)**  1.02 ( .69-1.52)  **.34 ( .17- .66)** | 1.00  .80 ( .42-1.52)  1.13 ( .64-1.99)  .63 ( .36-1.08)  .69 ( .34-1.08) |
| Educational level mother  High (ref.)  Middle  Low | 1.00  **1.43 (1.05-1.93)**  1.02 ( .61-1.71) | 1.00  .79 ( .54-1.18)  .70 ( .36-1.35) |

Bold: represents p≤0.05. OR=Odds Ratio for psychosocial care use at age 9 years. 95% CI=95% confidence interval. ^a^Total problem score on Child Behavior Checklist in the borderline or clinical range. ^b^Score on Child Health Questionnaire (ranging from 0-100). Adjusted for Age at visit research centre. Interaction term ‘presence of emotional/behavioural problems at 5 years *gender’, added to the multivariable analysis of Table 2, had a p-value of .102.

|  | Multivariable analysis^a^, stratum: Boys  (N=2,322, care use N=260 (11.1%)) | Multivariable analysis^a^, stratum: Girls  (N=2,392, care use N=164 (6.9%)) |
| --- | --- | --- |
| *Type of problem at age 5 years old* | *OR (95% CI)* | *OR (95% CI)* |
| Emotionally reactive  Anxious depressed  Somatic complaints  Withdrawn  Sleep problems  Attention problems  Aggressive problems | 1.34 ( .78-2.31)  1.40 ( .89-2.22)  1.26 ( .83-1.90)  **1.43 (1.06-1.94)**  1.09 ( .73-1.64)  **1.53 (1.07-2.18)**  **2.48 (1.36-4.51)** | 1.42 ( .70-2.90)  1.40 ( .82-2.39)  1.30 ( .82-2.08)  1.15 ( .77-1.71)  .99 ( .62-1.60)  1.46 ( .85-2.51)  1.20 ( .43-3.33) |
| Externalising scale  Internalising scale | **2.11 (1.41-3.16)**  **2.17 (1.49-3.16)** | 1.21 ( .60-2.41)  **1.87 (1.17-2.99)** |
| *Type of problem at age 9 years old* | *OR (95% CI)* | *OR (95% CI)* |
| Anxious/depressed  Withdrawn/depressed  Somatic complaints  Social problems  Thought problems  Attention problems  Rule breaking  Aggressive behaviour | **2.20 (1.27-3.80)**  **1.68 (1.11-2.55)**  .78 ( .45-1.37)  1.17 ( .66-2.06)  **2.14 (1.31-3.50)**  **3.41 (2.15-5.41)**  .71 ( .27-1.87)  1.70 ( .96-3.03) | **3.06 (1.70-5.30)**  1.26 ( .61-2.58)  1.16 ( .66-2.03)  1.45 ( .67-3.16)  1.48 ( .82-2.69)  **3.78 (2.17-6.58)**  1.82 ( .74-4.49)  1.05 ( .44-2.52) |
| Externalising scale  Internalising scale | **2.84 (1.98-4.06)**  **2.70 (1.96-3.71)** | **3.63 (2.17-6.07)**  **2.70 (1.77-4.09)** |

Appendix Table S IV, Associations between types of emotional/behavioural problems or externalising and internalising problems at 5 and 9 years old, and psychosocial care use at 9 years old, stratified by gender (N=4,714)

Bold:represents p≤0.05. OR=Odds Ratio for psychosocial care use at age 9 years. 95% CI=95% confidence interval. ^a^Adjusted for the other syndrome/broadband scales, sex, ethnic background mother, educational level mother, age at visit research centre. The problems represent the presence of borderline/clinical problems on the syndrome/broadband scales of the Child Behavior Checklist. The syndrome scales and broadband scales are analysed in separate models. Interaction term ‘presence of emotional/behavioural problems at 5 years *gender’, added to the multivariable analysis of Table 2, had a p-value of .102.
